# Advances in understanding and treating diabetic kidney disease: focus on tubulointerstitial inflammation mechanisms

**DOI:** 10.3389/fendo.2023.1232790

**Published:** 2023-10-04

**Authors:** Chengren Xu, Xiaowen Ha, Shufen Yang, Xuefei Tian, Hong Jiang

**Affiliations:** ^1^ Division of Nephrology, Department of Internal Medicine, People’s Hospital of Xinjiang Uygur Autonomous Region, Urumqi, China; ^2^ Section of Nephrology, Department of Internal Medicine, Yale University School of Medicine, New Haven, CT, United States

**Keywords:** diabetic kidney disease, tubulointerstitium, inflammatory mechanism, therapeutic targets, signaling pathway

## Abstract

Diabetic kidney disease (DKD) is a serious complication of diabetes that can lead to end-stage kidney disease. Despite its significant impact, most research has concentrated on the glomerulus, with little attention paid to the tubulointerstitial region, which accounts for the majority of the kidney volume. DKD’s tubulointerstitial lesions are characterized by inflammation, fibrosis, and loss of kidney function, and recent studies indicate that these lesions may occur earlier than glomerular lesions. Evidence has shown that inflammatory mechanisms in the tubulointerstitium play a critical role in the development and progression of these lesions. Apart from the renin-angiotensin-aldosterone blockade, Sodium-Glucose Linked Transporter-2(SGLT-2) inhibitors and new types of mineralocorticoid receptor antagonists have emerged as effective ways to treat DKD. Moreover, researchers have proposed potential targeted therapies, such as inhibiting pro-inflammatory cytokines and modulating T cells and macrophages, among others. These therapies have demonstrated promising results in preclinical studies and clinical trials, suggesting their potential to treat DKD-induced tubulointerstitial lesions effectively. Understanding the immune-inflammatory mechanisms underlying DKD-induced tubulointerstitial lesions and developing targeted therapies could significantly improve the treatment and management of DKD. This review summarizes the latest advances in this field, highlighting the importance of focusing on tubulointerstitial inflammation mechanisms to improve DKD outcomes.

## 1 Introduction

Diabetes is a global chronic disease that is experiencing a rise in its incidence (1). Recent data from the International Diabetes Federation (IDF) predict that by 2045, more than 10.5% of adults worldwide will be affected by diabetes, leading to kidney damage in 100 to 250 million individuals (2, 3). Formerly, the prevailing notion was that diabetes, particularly type 2 diabetes, predominantly affected the glomerular structures of the kidneys, a condition termed diabetic nephropathy (DN) (4). However, as our understanding of the pathogenesis, clinical presentation, and pathological patterns of kidney injury in diabetes improved, the National Kidney Foundation Kidney Disease Outcomes Quality Initiative (NKF/KDOQI) guidelines introduced the term “Diabetic kidney disease “ in 2007 to replace diabetic nephropathy (5) Tervaert and colleagues reported in 2010 that some individuals with diabetes and kidney involvement might not display clinical signs of albuminuria (6).However, these patients exhibit kidney impairment and/or clinical manifestations of tubular dysfunction, such as renal tubular acidosis. Renal biopsy results revealed a predominant involvement of tubules, interstitium, and/or blood vessels, accompanied by relatively uncharacteristic glomerular changes like thickened glomerular basement membranes, expansion of the mesangial matrix, and glomerulosclerosis (6). Follow-up studies have substantiated these pathological findings (7–9). Even in DKD patients primarily manifesting glomerular lesions, renal biopsies unveiled varying extents of tubulointerstitial injury (10, 11).These findings prompted a consensus between the American Diabetes Association (ADA) and the NKF to adjust the terminology from “diabetic nephropathy “ to “diabetic kidney disease” with the intention to more accurately describe the spectrum of kidney impairment caused by diabetes (12, 13). This alteration underscores the evolving understanding and recognition of the intricate role of kidney involvement in the context of diabetes.

Previously, DKD was thought to primarily involve glomerular pathological changes (14). However, recent research has unveiled a strong association between the extent of tubulointerstitial injury and the progression of kidney function as well as the prognosis (7, 15). Tubulointerstitial lesions can occur independently of glomerular lesions (9, 16, 17). Clinical investigations have proposed that the decline in glomerular filtration rate (GFR) in DKD patients devoid of proteinuria is mainly attributed to tubulointerstitial injury (18). Vascular endothelial growth factor (VEGF) activation coupled with diminished nitric oxide (NO) levels can induce vasoconstriction in small blood vessels (19). Elevated blood pressure and suboptimal blood glucose control, resulting in mechanical stress, contribute to reduced peritubular blood flow, exacerbating hypoxia (7, 19). These findings collectively suggest that harm to proximal tubular epithelial cells (PTECs)not only affects their functioning but also extends to more extensive glomerular harm, encompassing podocyte injury (9, 20). Consequently, tubular injury could manifest prior to glomerular damage, which warrants further investigation.

The precisemechanisms underlying tubulointerstitial lesions in DKD are not fully understood. However, immune inflammation is recognized as a characteristic feature associated with the development and progression of tubular injury in DKD (21–23) In the early stages of the disease, there is hypertrophy and an increased number of tubular epithelial cells, as well as thickening of the tubular basement membrane. These factors are believed to be critical in initiating and promoting the process of tubulointerstitial fibrosis (11). The prolonged presence of high blood glucose levels, along with ischemia and hypoxia, leads to tubular cell apoptosis, tubular atrophy, and degeneration (24) Inflammatory cell infiltration, increased production of inflammatory factors, interstitial fibrosis, arteriosclerosis of the interstitial small arteries, and hyaline changes in the arterioles also contribute to the occurrence and progression of the lesions (4, 6).

The existing treatments such as glycemic control, blockade of the renin-angiotensin-aldosterone system, stabilization of hemodynamics, and prevention and management of complications are not able to completely inhibit the progression of DKD, highlighting the urgent need for new therapeutic targets (4). In the past decade, apart from SGLT-2 inhibitors, mineralocorticoid receptor antagonists (MRAs) have also shown promising results as a class of drugs (25). The new generation of nonsteroidal selective MRA, finerenone, can prevent various injuries caused by excessive MR activation, inflammatory diseases, and fibrosis processes, thereby promoting the recovery of cardiac and kidney function (26, 27). Since 2021, finerenone has been approved by the U.S. Food and Drug Administration(US FDA), the European Medicines Agency (EMA), and the Chinese National Medical Products Administration (NMPA) for use in patients with DKD and patients with nondiabetic kidney disease (NDKD) accompanied by chronic inflammation and interstitial fibrosis.

In-depth exploration of the immunoinflammatory mechanisms underlying tubulointerstitial lesions in DKD and potential targeted therapies is of great clinical significance for the treatment of DKD. This review will discuss the immunoinflammatory mechanisms of tubulointerstitial lesions caused by diabetes and their relationship with potential targeted therapies. Additionally, the review will provide a detailed overview of the pathogenesis of tubular lesions in DKD and the use of medications, aiming to offer new insights for the development of DKD treatment strategies.

## 2 Mechanisms of renal tubular injuries in DKD

**Figure 1:**
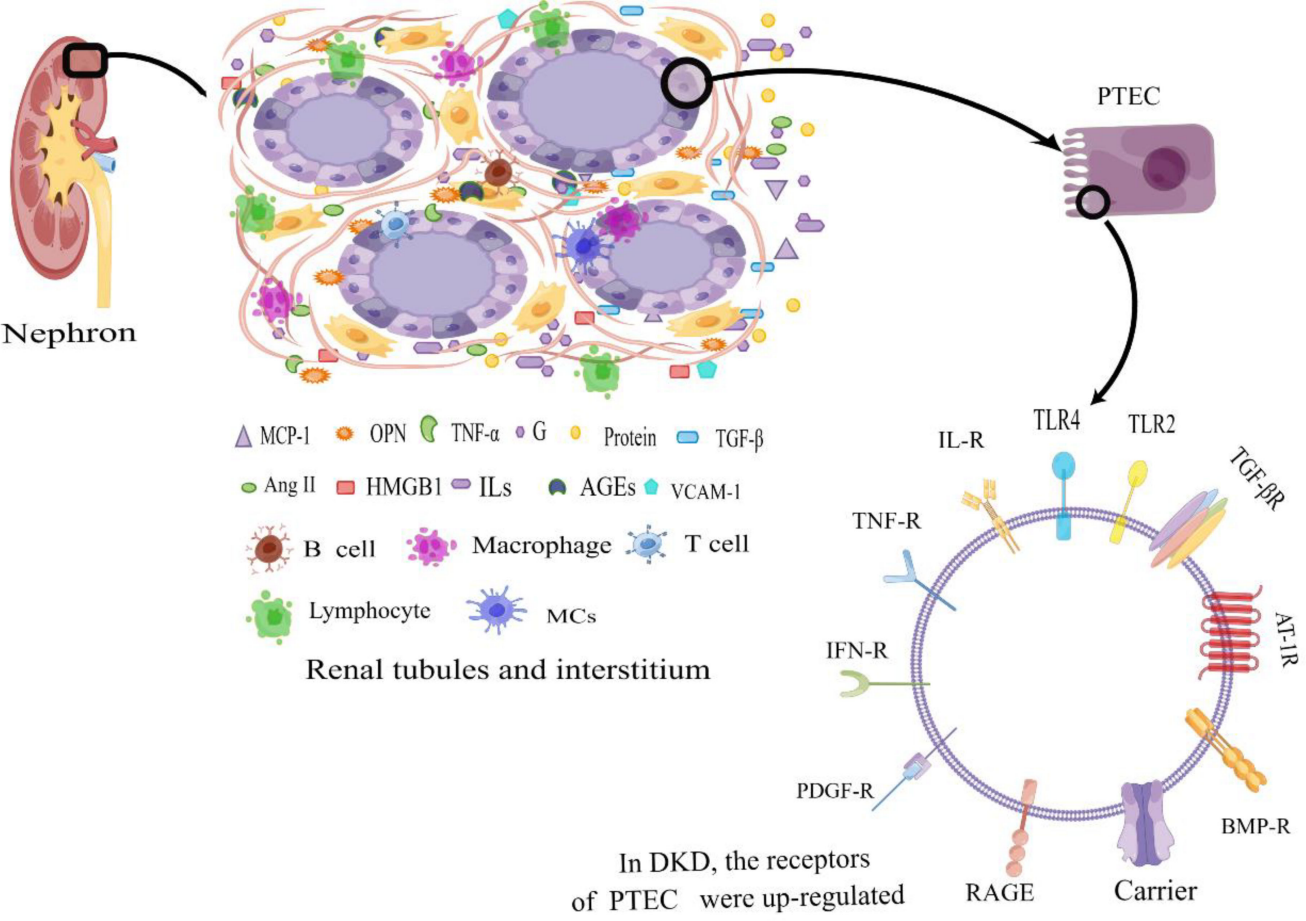
Diabetic tubular and interstitial inflammatory states. In DKD patients, in the tubulointerstitium, excessive protein and a high glucose environment lead to an inflammatory state of renal tubular epithelial cells. The generated large amount of pro-inflammatory cytokines attract inflammatory cells, which secrete a significant amount of inflammatory cytokines. The accumulation of inflammatory factors at the site of injury leads to tubular atrophy, interstitial inflammation, and fibrosis. Additionally, the upregulation of surface receptors on renal tubular epithelial cells further amplifies the inflammatory response, contributing to the progression of DKD. PTEC, Renal Tubular Epithelial Cells; IL-R, Interleukin Receptor; TLR2, Toll-like Receptor 2; TLR4, Toll-like Receptor 4; TNF-R, Tumor Necrosis Factor Receptor; IFN-R, Interferon Receptor; RAGE, the Receptor of Advanced Glycation Endproducts; PDGF-R, Platelet-derived Growth Factor Receptor; BMP-R, Bone Morphogenetic Protein Receptor; AT-1R, Angiotensin Receptor 1; TGF-βR, Transforming Growth Factor-β Receptor; MCP-1, Monocyte Chemotactic Protein 1; OPN, Osteopontin; TNF-α, Necrosis Factor-α; G, Glucose; IFN-γ, Interferon γ; TGF-β, Transforming Growth Factor-β; Ang II, Angiotensin II; HMGB1, High-Mobility Group protein B1; ILs, Interleukin; AGEs, Advanced Glycation Endproducts; VCAM-1, Vascular Cell Adhesion Molecule 1; MCs, Mast Cells; By Figdraw (www.figdraw.com).

The renal tubules and interstitium constitute more than 90% of the kidney’s volume and serve a vital role in functions such as reabsorption, secretion, and excretion (28). Among these functions, the proximal renal tubular epithelial cells play a crucial role in reabsorbing almost all filtered glucose, proteins, and electrolytes from the glomerulus. Adequate blood supply and oxygen delivery are essential for maintaining their proper functionality (29). Impairment of renal tubules can lead to the leakage of substances like glucose, amino acids, and proteins into the urine, resulting in disruptions in urine concentration and dilution, electrolyte imbalances, and disturbances in acid-base equilibrium, among other clinical manifestations (30, 31). Some studies propose that the initial cause of microalbuminuria in DKD might stem from reduced albumin reabsorption capacity of proximal renal tubular epithelial cells, rather than originating from glomerular damage (32, 33). In this review, our primary focus is on the inflammatory mechanisms associated with renal tubular lesions. The initiation of sustained high glucose(HG) levels triggers a cascade of events involving inflammatory cells and factors, leading to the upregulation of surface receptors on renal tubular epithelial cells and thereby accelerating the progression of DKD (**Figure 1**).

### 2.1 Mechanisms of metabolic disorders

#### 2.1.1 Hyperglycemia

Hyperglycemia acts as the initiating factor causing tubulointerstitial damage in DKD (34). During the early stages of DKD, in response to the hyperglycemic environment, tubular epithelial cells undergo proliferation, hypertrophy, and experience excessive glucose and sodium reabsorption. This disruption in normal function leads to the dysregulation of the tubuloglomerular feedback mechanism, resulting in intraglomerular high pressure, heightened perfusion, and increased filtration rates (35). Moreover, elevated glucose levels contribute to inflammatory responses: 1) The direct exposure of renal tubular cells to high glucose prompts the production of pro-inflammatory factors, including intracellular adhesion molecules such as Intercellular Adhesion Molecule 1 (ICAM-1), chemokines such as monocyte chemoattractant protein-1 (MCP-1), and osteopontin (OPN). These pro-inflammatory factors attract inflammatory cells, inciting their infiltration into the renal tubulointerstitium. This, in turn, triggers the release of an array of inflammatory mediators, leading to kidney tissue damage and actively participating in the progression of DKD (36, 37). Notably, among these factors, OPN emerges as a key chemokine responsible for inducing the infiltration of monocytes/macrophages into the tubulointerstitium (38). Histopathological examinations of renal biopsies from individuals with DKD consistently demonstrate the prevalence of macrophages among the infiltrating leukocytes within diabetic kidneys. The aggregation of macrophages within the tubulointerstitium emerges as a significant prognostic marker, reflecting the severity of renal impairment and the extent of interstitial fibrosis (39). It is worth noting that macrophage accumulation within the interstitial space, rather than within the glomeruli, is closely linked to proteinuria and the progressive decline in renal function. Furthermore, the extent of interstitial infiltration correlates proportionally with the rate of deterioration in kidney function (40).

Studies have demonstrated an upregulation of CD40 and CD40L expression in both tubular epithelial cells and macrophages within the context of DKD. This phenomenon has been substantiated by findings derived from renal biopsy pathology and cell-based experiments (41). In the presence of hypoxic conditions, the expression of CD40 within tubular epithelial cells triggers the secretion of Interleukin 6 (IL-6), thereby facilitating the interaction between inflammatory cells within the renal interstitium and the renal tubules (42). Notably, CD40 signaling in macrophages augments the production of pro-inflammatory and profibrotic mediators, contributing to tubular damage (43). Upon stimulation, CD40 receptors engage various tumor necrosis factor receptor-associated factors (TRAFs), initiating signaling cascades that encompass phosphoinositide 3-kinase (PI3K), Nuclear factor kappa-B (NF-κB), and p38/extracellular signal-regulated kinase (ERK) pathways (44). The signaling molecules activated by CD40 exhibit a dual nature, influencing both pro-inflammatory and anti-inflammatory responses (45). Substituting specific peptides within CD40L brings about selective modifications to CD40’s signaling and effector capabilities. This results in the induction of cytokine and enzyme expression, contributing to inflammatory reactions. Concurrently, this signal conversion can elevate the synthesis of anti-inflammatory cytokines. The distinct functional alterations observed in macrophage residues underscore their pleiotropic roles in this context (45). However, the precise mechanistic actions of CD40 within the DKD milieu, including the specific pathways it engages, remain incompletely understood and necessitate further exploration. A deeper comprehension of CD40’s role in DKD pathogenesis holds potential for unveiling therapeutic targets of significance.

To adapt to the HG environment, renal tubular cell surface receptors are upregulated. Specifically, in cultured PTECs, exposure to HG levels prompts the expression of toll-like receptor 4 (TLR 4) and triggers the release of the endogenous TLR ligand high mobility group box-1 (HMGB-1) from tubular epithelial cells and podocytes, mediated by Protein kinase C (PKC) activation (46). This combination of factors culminates in the activation of NF-κB and the subsequent upregulation of IL-6 and C-C Motif Chemokine Ligand 2 (CCL-2)/MCP-1 expression (47). Notably, TLR4 exhibits heightened expression within renal tubules, displaying a positive correlation with interstitial macrophage infiltration and Glycated hemoglobin (HbA1c) levels, while manifesting a negative correlation with the estimated GFR as determined during renal biopsy (48). Moreover, the TLR4-mediated pathway has shown potential to promote tubulointerstitial inflammation in DKD (46). In a similar vein, TLR2 has been implicated in stimulating the increased production of pro-inflammatory cytokines through the mitogen-activated protein kinase (MAPK), P38, and ERK pathways in various disease contexts (49, 50). In the context of streptozotocin-induced diabetic rats, tubular TLR2 expression witnesses a significant upregulation, coupled with heightened renal expression of MCP-1 and myeloid differentiation factors such as Myeloid differentiation primary response 88 (MyD88). This is accompanied by NF-κB activation, macrophage infiltration, and the presence of endogenous ligands like heat shock protein 70(HSP70) and HMGB1 that interact with TLRs (47). Notably, the activation of TLR2, TLR4, and Advanced glycosylation end product-specific receptor (RAGE) serves as a pivotal mechanism through which damage-associated molecular patterns (DAMPs) contribute to immune-mediated injury in DKD (51).

Conventionally, it is recognized that hyperglycemia induces the glycosylation of complement regulatory proteins, subsequently impairing their regulatory capacity (52). Interestingly, the blockade of complement or MCP-1 activity has exhibited efficacy in ameliorating tubulointerstitial injury (53). Consequently, it is postulated that the damage to tubular epithelial cells might be exacerbated by increased concentrations of activating components within the complement system and its associated endogenous regulatory proteins (such as C3, C3a, C5a, C5b–9, and Crry) (54, 55). Additionally, certain components of the complement system possess chemotactic properties, thus potentially inciting an inflammatory response that inflicts damage upon the tubulointerstitium. The release of biologically active substances, including trypsin, chymotrypsin, transforming Growth Factor-β1 (TGF-β1), renin, and Tumor Necrosis Factor- alpha (TNF-α), into the tubulointerstitium via degranulation of mast cells further accentuates renal inflammation (56). Furthermore, the complement pathway (C3a) and the TLR pathway associated with microbial pattern recognition receptors are additional conduits through which renal inflammation can be instigated (57).

Elevated glucose levels play a pivotal role in the promotion of tubular and interstitial fibrosis. Under the influence of HG and other stimulating factors, PTECs undergo a phenotypic transformation, adopting a myofibroblast-like phenotype within the tubulointerstitium. Simultaneously, these cells produce profibrotic factors, such as TGF-β1, which contributes to the synthesis of extracellular matrix (ECM) and exacerbates renal damage (58). Notably, TGF-β emerges as a crucial cytokine in driving the phenotypic transformation of epithelial cells, with other cytokines, such as Interleukin-1 (IL-1), regulating the production of TGF-β (59). TGF-β1 stimulates an increase in the synthesis of various ECM components, including lamin, fibronectin (FN), and type IV collagen, culminating in the excessive production of ECM. Furthermore, TGF-β1 hinders the expression and activity of matrix metalloproteinases (MMPs), which are pivotal for ECM degradation, while simultaneously augmenting the expression and activity of MMP inhibitors. Consequently, these actions collectively contribute to the development of tubulointerstitial fibrosis (60). TGF-β1 additionally propels renal cell hypertrophy and ECM accumulation, serving as a key conduit that leads to tubulointerstitial fibrosis (61). Within the renal interstitium, Platelet-Derived Growth Factor Receptor-alpha (PDGFr-α) is constitutively expressed. The expression of its ligand, PDGF-CC, is induced through the infiltration of monocytes and macrophages. This ligand, in turn, triggers a cascade of signaling pathways encompassing Janus Kinase/Signal Transducers and Activators of Transcription (JAK/STAT), PI3K, Phospholipase C-γ(PLC-γ), and MAPK, ultimately regulating gene expression. These intricate pathways enhance fibroblast proliferation, migration, and ECM synthesis (62, 63). Mice-based experiments have substantiated that the preferential expression of PDGF-¬DD in interstitial fibroblasts is evident during both the early and late stages of renal inflammation. Additionally, PDGF-CC within the peritubular capillary endothelium exerts its profibrotic impact by directly inducing fibroblast proliferation and enhancing leukocyte infiltration, collectively culminating in the development of tubulointerstitial fibrosis (64, 65). Moreover, HG levels stimulate pro-inflammatory, profibrotic, and angiogenic signaling within the tubules. This prompts the production of IL-6, CCL-2, and TGF-β, with a portion of this response mediated through the Bradykinin (BK)-mediated MAPK p42/p44 signaling pathway (37)..

#### 2.1.2 Advanced glycation end products

AGEs are formed through non-enzymatic reactions between reducing sugars and proteins, amino acids, and other molecules (66).. Notably, the proximal tubule serves as the principal site for AGE reabsorption (67). The presence of AGEs triggers the expression of Interleukin-8 (IL-8) and soluble ICAM-1 in PTEC. This, in turn, fosters the infiltration of inflammatory cells into the tubulointerstitial space (68). AGEs are also capable of activating CD4+ and CD8+ T cells, which infiltrate the mesenchyme and secrete cytokines such as Interferon-gamma (IFN-γ) and TNF-α. This inflammatory response, accompanied by oxidative stress, contributes to tissue inflammation within the renal environment. Consequently, this inflammatory milieu leads to compromised function of the glomerular capillary wall barrier and increased albumin permeability (69, 70). Furthermore, AGEs initiate activation of the PKC pathway, subsequently inducing the expression of inflammatory mediators, including ICAM-1, Vascular Cell Adhesion Molecules (VCAM-1), and MCP-1. This observation implies a pivotal role for PKC in the context of DKD (71, 72).

In the context of diabetes, the number of binding sites for AGEs on the proximal renal tubules increases (66). Upon binding to their receptors, AGEs can induce the production of TGF-β1 (73). Once bound to the receptor, active TGF-β1 is translocated to the nucleus by recruiting and phosphorylating Smads, specifically Smad2 and Smad3, thereby regulating the expression of genes associated with collagen production (74). Bone Morphogenetic Protein-7 (BMP-7) serves as a natural antagonist of TGF-β and possesses the capacity to counteract renal tubulointerstitial fibrosis by engaging the Smad1/5 pathway. This involves the formation of complexes with Smad4, nuclear translocation, and the phosphorylation of Smad3 (75, 76). Furthermore, it’s worth noting that Smad-independent signaling pathways associated with TGF-β are also implicated, including the activation of p38 MAPK, c-Jun N-terminal Kinase (JNK), and Rho (77). AGEs stimulate the expression of Connective Tissue Growth Factor (CTGF) in renal tubular epithelial cells. The ERK/p38-Smad3 signaling pathway interacts with the Epidermal growth Factor Receptors(ERGF)/p38-Smad3 signaling axis, contributing significantly to renal tubulointerstitial fibrosis. Importantly, this mechanism operates independently of the canonical TGF-β signaling pathway (78).

#### 2.1.3 Fatty acid metabolism

Tubular epithelial cells exhibit a preference for energy production through fatty acid oxidation (FAO) as opposed to glucose metabolism (79). The mitochondrial oxidative activity of fatty acids profoundly influences tubular reabsorption (80). Consequently, lipid metabolism and the maintenance of proximal tubular mitochondrial function hold immense significance within renal tubular cells. The detrimental impact of lipotoxicity expedites the decline of tubular function, emphasizing the critical role of lipid deposition in the renal tubules, which is elucidated as follows:

1) CD36-Mediated Lipid Deposition: The transmembrane glycoprotein CD36, responsible for long-chain fatty acid transport, significantly influences lipid accumulation in tissues (81). Peroxisome proliferator-activated receptor γ (PPAR γ), a ligand-activated transcription factor, governs CD36 expression and functionality (82). Research demonstrates that HG activates the protein kinase B/(AKT)-PPARγ signaling pathway, resulting in an upsurge of CD36 mRNA and protein within tubular epithelial cells. This elevated CD36 expression enhances cellular uptake of free fatty acids, ultimately contributing to diminished tubular cell viability and lipid deposition (83). Targeting the inhibition of AKT-PPARγ pathway could hold therapeutic potential for curtailing lipid accumulation in DKD.
2) Nϵ-carboxymethyl lysine (CML)and RAGE-Mediated Lipid Accumulation: CML, an AGE, plays a role in lipid homeostasis (84). CML binds to its receptor RAGE, which is expressed on the surface of renal tubular epithelial cells. This interaction triggers intracellular signaling pathways that heighten cholesterol synthesis via 3-hydroxy-3-methylglutaryl-coenzyme A reductase (HMG-CoAR) and augment low-density lipoprotein receptor (LDLr)-mediated cholesterol uptake through endoplasmic reticulum stress (ERS). Concomitantly, CML diminishes ATP-binding cassette transporter A1 (ABCA1)-mediated cholesterol efflux, collectively driving lipid accumulation within HK-2 cells. This process eventually culminates in tubular foam cell formation (85).
3) Phosphofluoric acid cluster sorting protein 2(PACS-2) and Tubular Lipid Accumulation: PACS-2, a multifunctional sorting protein predominantly expressed in renal tubules, plays a pivotal role in lipid metabolism (86). In studies involving streptozotocin-induced diabetic mice and DKD patients, reduced levels of PACS-2 expression have been observed. Notably, diabetic tubule-specific Pacs-2 deletion accentuates lipid synthesis through upregulated sterol O-acyltransferase 1 (SOAT1) expression and concurrent inhibition of cholesterol efflux (87). This intensified lipid accumulation in tubular cells contributes to the progression of albuminuria excretion and exacerbates kidney damage.

In summary, the accumulation of intracellular lipids can initiate a cascade of events including ERS, heightened production of reactive oxygen species (ROS), and the instigation of inflammatory responses (88). The phenomenon of lipotoxicity engenders mitochondrial dysfunction, prompting renal tubular epithelial cells to adopt fibrotic phenotypes characterized by augmented ATP consumption, cellular demise, dedifferentiation, and the internal deposition of lipids. This collective process ultimately culminates in the development of tubulointerstitial fibrosis (89).

### 2.2 Proteinuria

Under normal physiological conditions, the proteins that are filtered from the glomerulus undergo near-complete reabsorption in the proximal tubules. In the context of DKD, the occurrence of microalbuminuria may be attributed to the impaired ability of tubules to reabsorb albumin, rather than being primarily caused by glomerular damage The elevated quantity of filtered proteins serves as a stimulus for the signaling pathway of PTECs, leading to abnormal regulatory responses that encompass phenomena like tubular cell growth, apoptosis, changes in gene transcription, and further induction of inflammatory factors that contribute to inflammation and fibrosis When examined from an inflammatory perspective, proteinuria emerges as a key pro-inflammatory stimulus in activating NF-κB within renal tubular cells (90). Chemokines and adhesion molecules, orchestrated through the NF-κB-dependent pathway, are also augmented due to excessive ultrafiltration proteins within proximal tubular cells. Thus, it can be posited that the activation of NF-κB and the transcription of specific pro-inflammatory chemokines constitute hallmarks of progressive DKD (91). A specific instance is the C-X3-C motif chemokine 1 (CX3CL1), a membrane-bound chemokine that prompts the upregulation of CX3CR1 in proximal tubular epithelial cells through the NFκB and p38–MAPK-dependent pathways (92). Similarly, the heightened expression of C-C motif chemokine 5 (CCL5, also known as RANTES) is evident in renal biopsy samples from patients with type 2 diabetes mellitus, predominantly observed in renal tubular cells (90, 93). An overload of proteins triggers the upregulation of CCL5, and the extent of CCL5 expression in tubular cells is directly linked to the volume of proteinuria and the infiltration of interstitial cells The combined effects of CX3CR1 and CCL5 contribute to interstitial inflammation and the progression of the disease by facilitating the recruitment and adhesion of monocytes, T cells, and natural killer cells within the peritubular interstitium (93). Notably, the research conducted by Galkina et al. demonstrates that the main site for T cell recruitment is the stroma, particularly emphasizing a substantial increase (6-10 fold) in CD4+, CD8+, and CD20+ cells within the interstitium. Furthermore, the abundance of CD4+ and CD20+ cells correlates with the degree of proteinuria (94). The infiltration of interstitial T cells significantly correlates with the extent of proteinuria, hinting at an underlying immunopathological mechanism that contributes to the progression of proteinuria and interstitial inflammation in DKD (95). Moreover, the study highlights that VCAM-1 is upregulated in infiltrating cells within the renal interstitium in mice (96). The levels of VCAM-1 correspond to the number of immune cells infiltrating the kidneys, and they are also associated with the severity and progression of proteinuria (97, 98). Additionally, urine protein can initiate damage to tubular cells by activating the complement system and stimulating tubular cells to generate reactive oxygen species (99).

From the perspective of promoting fibrosis, TGF-β is currently regarded as a pro-inflammatory and fibrotic mediator induced by exposure to albumin. When protein is present in the urine, it binds to its receptors, triggering the release of cytokines that further promote inflammation and fibrosis. This cumulative effect ultimately results in the decline of kidney function (100). The presence of excess protein in urine has been found to regulate the expression of TGF-β and stimulate the deposition of extracellular matrix ECM, thereby intensifying fibrotic responses (101). In mice induced with streptozotocin (STZ) and featuring knockout of protein kinase C-epsilon (PKC-ϵ), an increase in microalbuminuria, tubulointerstitial fibrosis, and mesangial dilation was significantly observed. This suggests that PKC-ϵ deletion-mediated renal fibrosis might involve the TGF-β1 signaling pathway (102). Another form of PKC, PKC-β, exhibits enhanced activity within renal tubules. Inhibiting PKC-β has been shown to reduce renal macrophage accumulation, the expression of inflammatory molecules (such as ICAM-1 and MCP-1), and tubulointerstitial damage (72). During instances of renal fibrosis injury, Klotho plays a role in inhibiting the TGF-β1-induced epithelial-mesenchymal transition (EMT) response in cultured cells. This action is characterized by reduced expression of epithelial markers, interstitial markers, and cell migration (103). Experimental studies have speculated that this effect may be due to secreted Klotho directly binding to type II TGF-β receptors, thereby impeding TGF-β1 from binding to cell surface receptors and consequently inhibiting TGF-β1 signaling. Additionally, secreted Klotho has been found to hinder the EMT response by inhibiting Wnt and IGF-1 signaling pathways (104). Serum soluble Klotho (sKlotho) levels exhibit a significant negative correlation with varying degrees of urine albumin in patients with type 2 diabetes mellitus(T2DM). This insight suggests that sKlotho might serve as a potential biomarker to predict the progression of kidney disease in the future (105).

Excessive protein in urine can lead to the upregulation of gene expression in renal tubular cells, resulting in the overexpression of various chemokines. This, in turn, causes the aggregation of immune cells such as monocytes and T cells within the tubulointerstitium. The release of ILs further attracts neutrophils to aggregate, and this inflammatory environment promotes the synthesis of fibrous molecules like angiotensin II(Ang II) and TGF-β. These processes collectively contribute to the degradation of the tubular basement membrane, facilitating the entry of inflammatory cells into the interstitium and the capillary space surrounding the renal tubule. Ultimately, this sequence of events induces the occurrence of fibrosis (106). In summary, excessive urine protein stimulates proximal tubular epithelial cells, establishing a connection between proteinuria, interstitial inflammation, and fibrosis.

### 2.3 Oxidative stress

Persistent hyperglycemia results in an increase in AGEs, gradual enhancement of tubular Na+-K+-ATPase activity, and abnormal epithelial cell metabolism. These factors contribute to mitochondrial dysfunction and the production of large amounts of ROS (80). Consequently, endothelial cells undergo damage, leading to the recruitment of inflammatory cells and inflammatory factors to the site of injury. This process triggers a tubulointerstitial inflammatory response (68). In the context of *in vivo* studies, overexpression of Klotho has been found to effectively reduce ROS expression. This reduction occurs through the mitigation of TNF-α stimulation in the kidneys. As a result, Klotho overexpression inhibits NF-kB activation and consequently suppresses the production of inflammatory cytokines, chemokines, and growth factors. This action effectively curtails the oxidative stress response. However, it’s important to note that Klotho expression is generally low in cases of DKD (104, 107). Recent research highlights CD36 as a potential key mediator of ROS production in chronic kidney disease. Blocking CD36 interrupts the high glucose-induced EMT in tubular epithelial cells. This interruption primarily occurs via the ERK1/2 and TGF-β1/Smad2 signaling pathways (108).

The diabetic environment significantly contributes to the production of various chemokines, including MCP-1, OPN, CCL-2, CX3CL1, INF-γ-inducible protein (CXCL10), and CCL5. These chemokines play a crucial role in attracting inflammatory cells (macrophages and T cells) to the renal tubules and interstitium, thus establishing an inflammatory cycle (109). The infiltration of inflammatory cells in the tubules can lead to ruptures and thickening of the tubular basement membrane, ultimately resulting in tubular atrophy during later stages. Infiltration of inflammatory cells in the renal interstitium leads to the release of pro-inflammatory, profibrotic, and antiangiogenic factors, ultimately causing interstitial fibrosis (IFTA). Interestingly, renal biopsy findings reveal the presence of interstitial eosinophil aggregates (IEAs) in patients with DKD (110). IEA’s severity correlates with IFTA, making it challenging to conclusively diagnose allergic interstitial nephritis. This indicates that the IEA in DKD might either reflect an inflammatory response to chronic tubulointerstitial injury, or potentially serve as a stimulus contributing to such injury, or even a combination of both factors. The published article primarily emphasizes tubulointerstitial inflammation, while further observation and study are required to fully comprehend tubulitis (111).

### 2.4 Mechanism of ischemia and hypoxia

The blood supply to tubular epithelial cells primarily originates from the efferent arterioles of the glomeruli, eventually reaching the veins surrounding the renal tubules. This process is intricately regulated by various neurohumoral factors. The damage caused by proximal tubular hypoxia in DKD can be attributed to three primary factors:

1) Decreased blood flow: The decrease in blood flow within peritubular capillaries (PTCs) mainly arises from dysregulation in vasoconstriction and relaxation factors. In the presence of AGEs and elevated glucose levels, the phosphorylation and expression of endothelial nitric oxide synthase (eNOS) decrease, leading to dysfunction and damage of vascular endothelial cells (112). NO, a vasoactive substance, plays a crucial role in promoting vasodilation and safeguarding vascular endothelial cells. However, its synthesis is reduced. Simultaneously, Ang II contributes to the constriction of both afferent and efferent arterioles. It promotes endothelial cell proliferation, hypertrophy, and reduces blood supply to the peritubular capillaries (113).
2) Impaired oxygen utilization: The process of sodium-glucose transport across the membrane of proximal tubular cells is not energy-intensive but relies on the activity of the Na+/K+ ATPase. In diabetic rats, impaired mitochondrial ATP production and fragmentation of organelles in proximal tubular epithelial cells have been observed at an early stage of the disease. This is associated with increased urinary albumin excretion, abnormal glomerular morphology, and even elevated urinary kidney injury molecule-1 (KIM-1) levels (114). These mitochondrial structural and functional abnormalities may represent the earliest manifestations of DKD (115). In patients with DKD, glucose reabsorption increases, metabolic activity rises, O2 consumption elevates, and mitochondrial structure and dysfunction lead to impaired oxygen utilization. As a result, the proximal tubule becomes more susceptible to ischemic injury and tends toward acute kidney injury (AKI) (116).
3) Microvascular thinning:Given the poor tolerance of renal tissue to hypoxia and the vital reabsorption function of renal tubules, the development of a complex and dense microvascular network is essential (117). In the context of DKD, an imbalance exists between pro-angiogenic and antiangiogenic factors. Animal studies reveal that levels of VEGF mRNA and protein decrease, while the expression of thrombospondin-1 (TSP-1), an inhibitor of angiogenesis, increases. These changes lead to endothelial swelling, capillary loss, and proteinuria (118). Hypoxia contributes to increased extracellular matrix production through both TGF-β-dependent and non-dependent mechanisms. Hypoxia-inducible factor (HIF) upregulates the expression of CTGF. This results in an increased diffusion distance for oxygen delivery to the parenchyma. The fibrotic expansion of the interstitium further compresses and disrupts the local microvascular network, ultimately causing microvascular rarefaction (9). This intensifies the degree of tubulointerstitial hypoxia, establishing a detrimental feedback loop.

Hypoxia is a crucial factor underlying the onset and progression of DKD. Hemodynamic shifts, metabolic influences, and immune triggers can directly harm vascular endothelial cells, leading to local activation of the RAS or a reduction NO levels. This prompts renal vasoconstriction and hampers oxygen delivery. As oxygen transport diminishes, it disrupts kidney perfusion, intensifying renal hypoxia. Consequently, renal tubular epithelial cells experience mitochondrial dysfunction and impaired oxygen utilization, resulting in cellular degeneration, atrophy, damage to periductal capillaries, and a subsequent reduction in blood supply. These cascading effects contribute to the development of interstitial fibrosis and a decline in kidney function.

## 3 Injurious biomarkers to renal tubular epithelial cells

### 3.1 Kidney injury molecule-1

KIM-1 is a glycoprotein expressed within the proximal tubules and serves as a sensitive indicator of tubular damage (119). While absent in normal kidneys, its expression significantly increases during acute injury or renal inflammation (120). KIM-1, found on PTECs, plays a role as a phagocytic cell, clearing cellular debris and apoptotic bodies within the damaged tubulointerstitial compartment. This action aids in the regeneration of injured tubules (121, 122). However, KIM-1 also facilitates the uptake of fatty acids by tubular cells, contributing to the progression of progressive DKD (123). The presence of KIM-1 correlates with tubulointerstitial inflammation and fibrosis (122). In patients with DKD, KIM-1 is strongly linked to the risk of progressive decline in kidney function, and its elevation has been observed in confirmed cases of DKD (124, 125). KIM-1 is relatively unstable, with its extracellular domain undergoing cleavage, shedding into the tubular lumen, and eventual excretion in the urine (126). In DKD, urinary KIM-1 levels suggest early tubular involvement (127). Currently, urinary KIM-1 is widely accepted as a specific and sensitive biomarker for assessing renal proximal tubular injury (128).

### 3.2 Neutrophil gelatinase-associated lipocalin

NGAL is a 25-kDa glycoprotein with widespread distribution in human tissue cells. Its expression in renal tissue is minimal under normal conditions. NGAL is primarily expressed in Henle’s loops and distal tubules, playing a role in iron metabolism by binding to iron transporters (129). NGAL promotes tubular epithelial cell regeneration by inducing apoptosis of infiltrated neutrophils within the tubulointerstitium, thereby protecting renal tissue from inflammatory cell damage (130). In cases of kidney ischemia and hypoxia, the expression of NGAL in tubular epithelial cells is significantly enhanced. Damaged tubular cells produce NGAL, which is subsequently secreted into the blood and urine (131). Urinary NGAL levels can detect AKI within 2 hours, while significant changes in serum creatinine take 3 to 4 days, underscoring NGAL’s role as an early and more sensitive indicator of AKI compared to serum creatinine (132). Research has also demonstrated a strong correlation between urinary NGAL levels and tubular atrophy (133). In cases of inflammatory injury, monocytes/macrophages, neutrophils, and tubular epithelial cells are the primary sources of NGAL production (127). Notably, tubular damage might manifest earlier than glomerular damage in DKD inflammatory states. As a marker of early tubular injury, NGAL can detect renal impairment in diabetic patients before the appearance of urinary microalbumin (mALB) (134).

**Figure 2:**
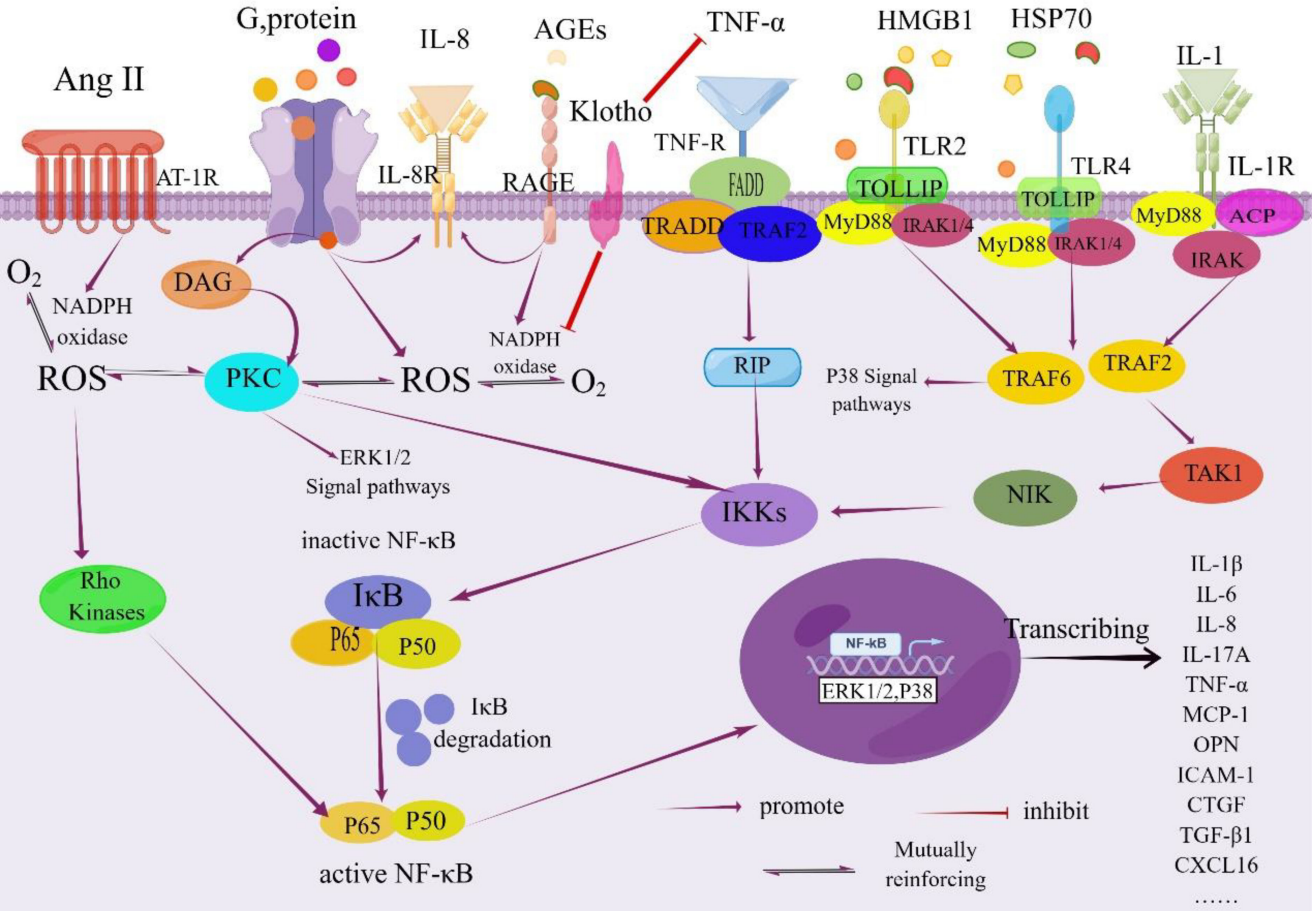
NF-κB signaling pathway in the pathogenesis of DKD. Activation of NF-κB is tightly regulated by the IκB regulatory protein family. This regulation occurs through the phosphorylation of the inhibitory protein IκB kinase by specific IκB proteins, followed by its degradation via proteolysis. Once freed, NF-κB translocates from the cytoplasm into the nucleus. Within the nucleus, NF-κB binds to specific promoter and enhancer sites on target genes, initiating the process of transcription. This activation leads to increased transcription of genes encoding inflammatory cytokines and other molecules associated with this complication As a result, renal inflammation is triggered due to the involvement of the NF-κB signaling pathway, ultimately contributing to the development of renal inflammation. IL-1R, Interleukin-1 Receptor; DAG, Diacylglycerol; PKC, Protein Kinase C; IkB, NF-kappa-B Inhibitor; FADD, Fas-associated with Death Domain Protein; TRADD, TNF Receptor 1 Associated via the Death Domain; TRAF2, TNF Receptor-Associated Factor 2; RIP, Receptor-interacting Protein; IKKs, IΚB Kinases; TOLLIP, Recombinant Toll Interacting IRAK, Interleukin Receptor-Associated Kinase; ACP, Acyl Carrier Protein; NIK, NF -κB Inducing Kinase; TAK1, Transforming Growth Factor Kinase 1; CTGF; Recombinant Connective Tissue Growth Factor; MyD88, Myeloid Differentiation Factor 88; HSP70, Heatshockprotein70; By Figdraw (www.figdraw.com).

In summary, hyperglycemia serves as the initiating factor for tubulointerstitial damage in DKD, triggering a cascade of cellular responses. This includes the accumulation of AGEs, oxidative stress activation, elevated intrarenal angiotensin II levels, and increased expression of pro-inflammatory and profibrotic factors. These processes collectively contribute to tubular atrophy, interstitial inflammation, and fibrosis, which are key factors in the progression of DKD (as depicted in **Figure 2**)

**Figure 3:**
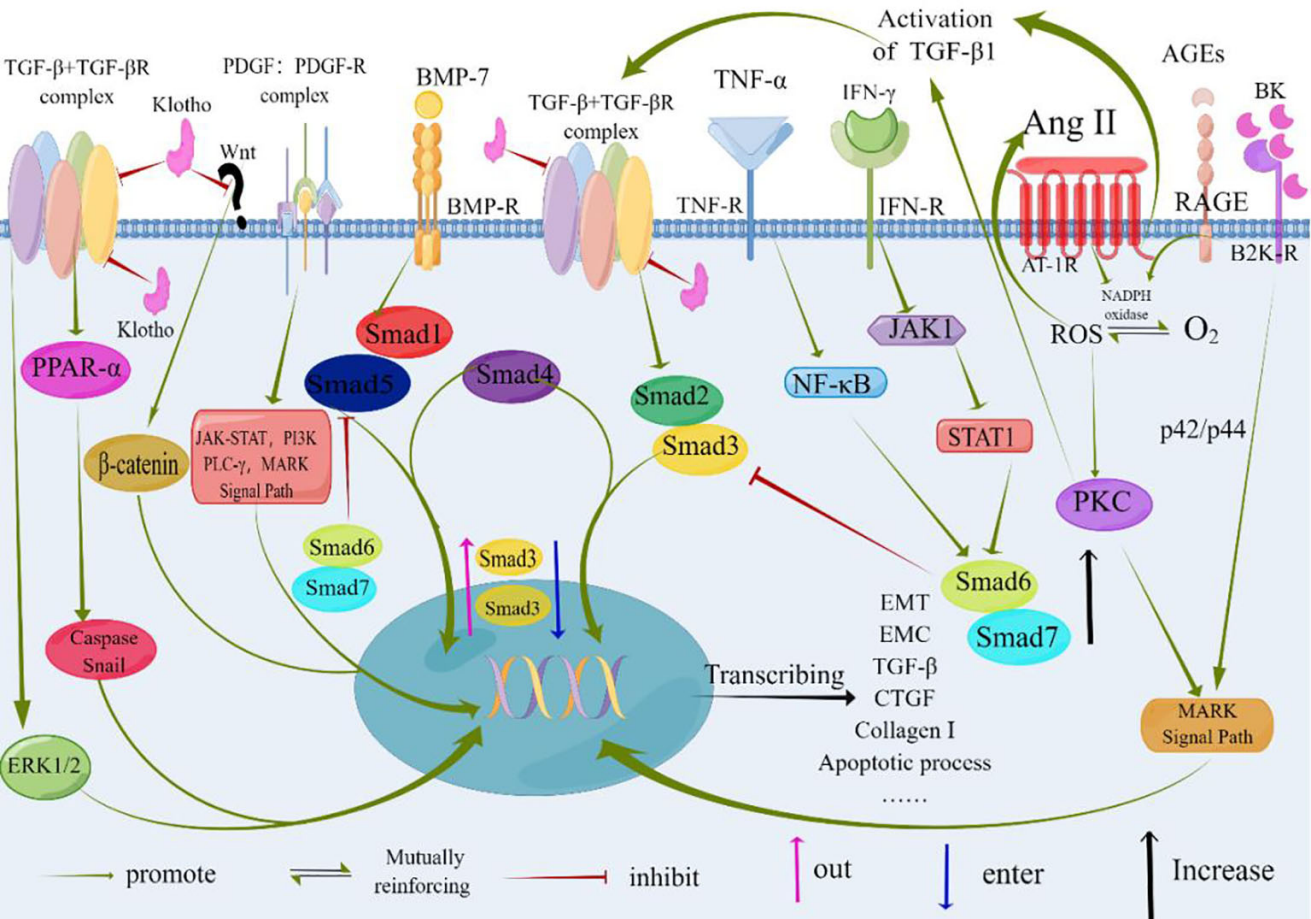
At the core of TGF-β signaling are pro-inflammatory and fibrotic processes. Factors such as albumin, hyperglycemia, and Angiotensinogen II contribute to renal interstitial fibrosis. TGF-β serves as a central mediator in these pro-inflammatory and fibrotic processes. Binding of TGF-β or BMP-7 to their respective receptors results in the formation of heteromeric receptor complexes with activated kinases (ALK5 and ALK3, respectively). This leads to the recruitment and phosphorylation of Smads (Smad2/3 for TGF-β and Smad1/5 for BMP-7). The phosphorylated Smads (pSmad) form heteromeric complexes with Smad4 and translocate to the nucleus, where they regulate gene expression. BMP-7 exerts antagonistic effects on TGF-β signaling, as depicted by its impact on the expression, nuclear shuttling, and phosphorylation of Smad3. Non-Smad signaling pathways of TGF-β can involve the activation of p38 MAPK, JNK, and Rho. Klotho inhibits the binding of TGF-β to cell surface receptors and blocks Wnt signaling, thereby inhibiting the fibrotic process and epithelial-mesenchymal transition (EMT). The specific molecular mechanisms underlying these effects need further investigation and confirmation. TGF-β, Transforming Growth Factor-β; TGF-βR, Transforming Growth Factor-β Receptor; PDGF, Platelet-derived Growth Factor; PDGF-R, Platelet-derived Growth Factor Receptors; BMP-7, Bone Morphogenic Protein-7; BMP-R, Bone Morphogenic Protein Receptor; AGEs, Advanced Glycation Endproducts; RAGE, the Receptor of Advanced Glycation Endproducts; Ang II, Angiotensin II; IFN-γ, Interferon γ; TNF-α, Tumor Necrosis Factor-α; AT-1R, Angiotensin Receptor 1; MAPK, Mitogen-activated Protein; PI3K, Phosphatidylinositol 3 Kinases; PLC-γ, Phospholipase C-γ; NF-kB, Nuclear Factor Kappa-light-chain-enhancer of activated B cells; JAK1, Janus Kinase 1; STAT1, Signal Transducer And Activator Of Transcription 1; PKC, Protein Kinase C; ROS, Reactive Oxygen Species; ERK1/2, Extracellular Signal-regulated Kinase 1/2; PPAR-α, Peroxisome Proliferators-Activated Receptor alpha; Smad, Drosophila Mothers Against Decapentaplegic Protein; CTGF, Connective Tissue Growth Factor; B2KR, Bradykinin 2- Receptor; BK, Bradykinin; EMT, Epithelial-to-Mesenchymal Transition; ECM, Extracellular Matrix; By Figdraw (www.figdraw.com).

Tubulointerstitial fibrosis is closely intertwined with the presence of infiltrating inflammatory cells. In response to these cells, stress signals, and various mediators—particularly TGF-β—tubular epithelial cells undergo a transformation into mesenchymal cells, a process known as epithelial-mesenchymal transition. Several factors, including excessive albumin, hyperglycemia, stimulation by angiotensin II, and activation of signaling pathways such as MAPK, JAK-STAT, and PKC, regulate the expression of TGF-β. Moreover, the Smad-independent TGF-β signaling pathway, which may involve p38 MAPK, JNK, and Rho activation, also plays a role. These intricate signaling pathways collectively impact gene expression, ultimately enhancing fibroblast proliferation, migration, and synthesis of the ECM (as depicted in **Figure 3** ).

## 4 Therapeutic targets for renal tubulointerstitial lesions

Following the approval of the first ACE inhibitor, captopril, by the US FDA in 1981 for reducing proteinuria and slowing the progression of kidney function, the exploration of novel RAS blockers has become a fundamental aspect of treating various kidney conditions. Initial investigations primarily centered on the kidney-protective benefits of RAS blockers through the enhancement of systemic blood pressure regulation and correction of abnormal renal hemodynamics (135–137). Nevertheless, starting from 2001, research unveiled that the RAS system also exhibits distinctive anti-inflammatory and CKD progression-inhibiting effects that are not solely tied to its blood pressure-lowering capabilities (138–141) In more recent years, as the understanding of the underlying mechanisms of DKD deepened and significant results from large-scale randomized controlled trials (RCTs) emerged, SGLT-2 inhibitors and non-steroidal mineralocorticoid receptor antagonists have emerged as effective therapeutic options for addressing DKD. These advancements have found widespread implementation in clinical practice.

### 4.1 SGLT-2 inhibitors

The protective effects ofSGLT-2i on the kidneys involve both hemodynamic and non-hemodynamic mechanisms. From a hemodynamic standpoint, SGLT-2i inhibits the function of SGLT-2 in the proximal tubules of the kidneys, reducing the reabsorption of sodium and glucose. This leads to increased sodium and glucose concentration in the thick ascending limb of the Loop of Henle, prompting juxtaglomerular cells to release renin. This process enhances tubuloglomerular feedback and induces constriction of the afferent arterioles. Consequently, the condition of high renal perfusion, pressure, and filtration in the glomeruli is improved, contributing to enhanced kidney function. This mechanism is pivotal in ameliorating the development and progression of DKD (142). In addition, SGLT-2i mitigates tubulointerstitial damage in DKD by exerting anti-inflammatory and antioxidative effects through the following mechanisms:

1) Angiotensin II regulation: Ang II enhances the expression of SGLT1 and SGLT2 in renal tubular epithelial cells, resulting in increased glucose uptake and elevated intracellular ROS levels, causing oxidative stress. SGLT2i effectively counteracts this heightened glucose uptake (143, 144).
2) Endothelial function: Ang II downregulates the function of eNOS, leading to endothelial dysfunction and a prothrombotic state. Reduced nitric oxide synthesis results in increased production of adhesion molecules, tissue factor, MCP-1, and β-galactosidase activity (145). SGLT2i facilitates AMPK phosphorylation, restores normal autophagy, reduces mammalian target of rapamycin (mTOR) activity, and diminishes the generation of inflammatory factors such as interleukin 1β (IL-1β), IL-6, and TNF-α (146). Furthermore, pentraxin-3 (PTX3), a protein promoting the M2 phenotype of macrophages that downregulates NF-κB, IL-1β, TNF-α, and MCP-1, is often significantly reduced in DKD patients (147, 148).
3) Epithelial-mesenchymal transition (EMT) inhibition: Under pro-inflammatory stimulation and excessive proteinuria, tubular epithelial cells undergo a transition to myofibroblasts. HG prompts the synthesis and release of matrix metalloproteinase 2 (MMP2), which degrades the tubular basement membrane, causing phenotypically altered tubular epithelial cells to detach and migrate to the interstitium. These cells, together with resident renal fibroblasts, subsequently produce excessive extracellular matrix proteins, leading to interstitial fibrosis. SGLT2i impedes the differentiation of endothelial progenitor cells (EPCs) or cells with EPC characteristics into myofibroblasts, thus notably reducing the number of cells undergoing EMT within the tubules (149, 150). Additionally, HIF-1α expression, prompted by hypoxia, fosters EMT and interstitial fibrosis by activating the TGF-β-dependent pathway and PI3K/Akt signaling pathway (151). Hence, the elevation of hemoglobin in DKD patients could also signify recovery from tubulointerstitial damage and fibrosis caused by HG (152, 153) SGLT2i effectively reduces the generation of microRNA-21 induced by high glucose, which targets RECK—a protein containing a Kazal motif that inhibits matrix metalloproteinase 2—thus suppressing EMT and cell migration. This represents another potential mechanism of renal protection (154).

Recent RCTs, including CREDENCE, DAPA-CKD, and EMPA-KIDNEY (**Supplementary Table 1**), have provided compelling evidence that the combination of SGLT2 inhibitors and RAS inhibitors in individuals with type 2 diabetes mellitus results in a decreased risk of kidney and cardiovascular endpoint events (155–160). Nevertheless, these investigations primarily emphasize clinical treatment outcomes and prognoses, lacking comprehensive insights into the progression of tubular and interstitial lesions.

### 4.2 Glucagon-like peptide-1 agonists

GLP-1 agonists, beyond their role in lowering glucose levels, exhibit renal protective effects encompassing anti-inflammatory, antioxidant properties, and a reduction in proteinuria. Studies in animal models reveal that GLP-1 agonists can diminish proteinuria and macrophage infiltration in mice, concurrently lowering levels of ICAM-1, TGF-β, and type IV collagen (161). The mechanism underlying these effects potentially involves restraining the production of inflammatory cytokines (IL-1β, TNF-α) through pathways influenced by Ang II and AGEs. This action leads to the inhibition of NF-κB, Rho-kinase activation, and signaling pathways like PKC and PKA, ultimately curbing inflammation and oxidative stress (162, 163).

In clinical studies (**Supplementary Table 1**), the administration of exenatide or liraglutide has demonstrated effective protection of the estimated GFR in diabetic patients, while also reducing microalbuminuria and diminishing urinary excretion of TGF-β1 and type IV collagen. This renal protective effect appears to be independent of glycemic control (164, 165)

### 4.3 Mineralocorticoid receptor antagonists

Aldosterone was initially identified for its action on the epithelial tissue of the distal tubules within the kidneys. This action, mediated by the mineralocorticoid receptor (MR), facilitates sodium reabsorption and potassium secretion, ultimately contributing to blood pressure elevation by expanding the extracellular volume (166). However, excessive MR receptor activity not only prompts sodium and water reabsorption, leading to elevated blood volume, but also triggers the generation of ROS and pro-inflammatory factors. These processes promote renal fibrosis, inflammatory responses, and kidney enlargement (167, 168). The occurrence of aldosterone escape following the use of ACE inhibitors or ARBs has prompted the application of aldosterone antagonists like spironolactone and eplerenone in the context of DKD treatment (169).

At the level of the cytoplasmic membrane, steroidal MRAs possess a chemical structure similar to aldosterone and compete for its binding sites, thereby exerting direct antagonistic effects. This action leads to the reduction of oxidative stress, inflammation, and the expression of pro-fibrotic factors. As a result, the inflammatory response and the progression of fibrosis are inhibited. To overcome the lack of selectivity in spironolactone and the lower affinity of eplerenone for MR, a newer generation of nonsteroidal MRAs, such as finerenone, has been developed. Finerenone’s distinct cellular mechanisms yield benefits like a lower incidence of treatment-related hyperkalemia and AKI, along with a milder impact on systolic blood pressure (170–172). Finerenone exhibits enhanced selectivity and receptor activity when compared to first- and second-generation mineralocorticoid receptor antagonists. Its advantageous features stem from its distinctive molecular structure and mode of action:

1) Chemical structure differentiation: Finerenone’s development builds upon the chemical structure of a dihydropyridine channel blocker, while lacking activity on L-type calcium channels. This characteristic grants it greater polarity than steroidal MRAs and enables its penetration of the blood-brain barrier, crucial for blood pressure regulation (173, 174).
2) Significant differences in tissue distribution pattern Finerenone demonstrates a balanced distribution across cardiac and renal tissues. In contrast, steroidal MRAs like spironolactone and eplerenone accumulate in the kidneys at levels at least three times higher. Consequently, finerenone more effectively reduces myocardial hypertrophy, plasma pro-brain natriuretic peptide levels, and proteinuria (175).
3) Distinct molecular receptor binding modes: With a sizable substituent, finerenone forms unstable MR-ligand complexes upon binding to MR. These complexes fail to recruit co-factors, differentiating its binding mode from other MRAs (174, 176, 177).

**Figure 4:**
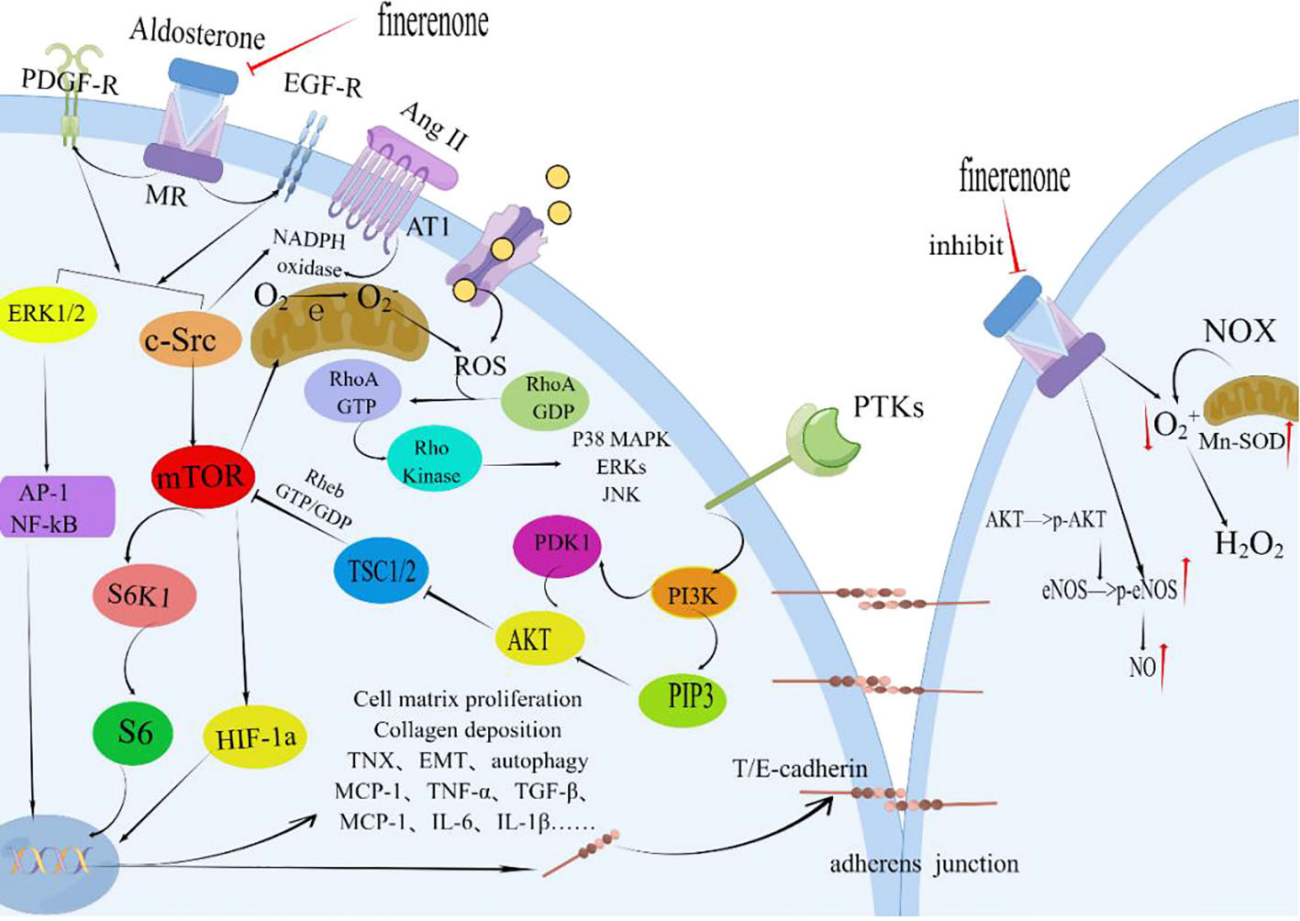
Finerenone improves the structure and function of renal tubular epithelial cells. By blocking the mineralocorticoid receptor (MR) and inhibiting the effects induced by the activation of MR by aldosterone, finerenone suppresses the actions of Ang II, PDGF, and EGF. It inhibits the phosphorylation of S6K1 through the ERK1/2 and c-Src/mTOR pathways, reducing oxidative stress and the generation of superoxide anions. Finerenone also increases the bioavailability of nitric oxide, repairs adhesion molecules, and improves the structural damage of proximal tubular cells (PTC). These mechanisms contribute to the maintenance of anti-inflammatory and anti-fibrotic effects in the kidneys. PDGF, Platelet-derived Growth Factor; PDGF-R, Platelet-derived Growth Factor Receptors; Ang II, Angiotensin II; AT-1R, Angiotensin Receptor1; MR, Mineralocorticoid Receptor; ERK1/2, Extracellular Signal-Regulated Kinase 1/2; AP-1, Activator Protein 1; c-Src, Non-receptor Receptor Tyrosine Kinase; EGFR, Epithelial Growth Factor Receptor; mTOR, Mammalian Target of Rapamycin; HIF-1a, Hypoxia-inducible Factor 1 alpha; PTKs, Protein Tyrosine Kinase; PI3K, Phosphatidylinositide 3-Kinases; PIP3, Phosphatidylinositol 3 Phosphate; AKT, Protein Kinase B(PKB); PDK1, 3-Phosphoinositide Dependent Protein Kinase-1; TSC1/2, Tuberous Sclerosis Complex1/2; NOX, NADPH Oxidase; Enos, endothelial Nitric Oxide Synthase; Mn-SOD, Mn-Superoxide Dismutase; TNX, Tenascin-X; EMT, Epithelial-to-Mesenchymal Transition; By Figdraw (www.figdraw.com).

Finerenone, through its MR-blocking action, contributes to the enhancement of renal tubular epithelial cell structure and function. This improvement is linked to diminished S6K1 phosphorylation and reduced oxidative stress. The primary effect is the inhibition of superoxide anion generation triggered by Ang II, PDGF, and EGF (**Figure 4**). This action facilitates the repair of adhesion molecules and ameliorates structural damage in proximal tubular cells(PTCs) (176, 178–180). In the early stages of interstitial fibrosis, the loss of the adhesion molecule-laminin complex contributes to disruption in PTC adhesion. The antagonistic impact of MR improves fibrosis-laminin-calcium-binding protein complexes by repairing adhesions. Notably, the MR system’s effects are contingent on factors like the ligand (aldosterone), cellular milieu, and target gene promoters. The MR, in conjunction with its co-regulatory factors, functions collaboratively in these pathways (181). Finerenone effectively delays the buildup of MR-aldosterone complexes and thwarts the recruitment of crucial transcription co-factors. Moreover, it successfully curbs the expression of tenascin-X (a recognized pro-fibrotic factor), enhances nitric oxide bioavailability, decreases ROS levels, mitigates endothelial dysfunction, myocardial hypertrophy, and proteinuria, and sustains anti-inflammatory and anti-fibrotic effects (176, 182).

In a distinct mechanism, a recent study published in JCI Insight revealed that MR antagonists have the capacity to reduce urinary protein excretion and impede DKD progression by safeguarding the glomerular endothelial glycocalyx (GEnGlx) (183). GEnGlx constitutes a hydrated polyanionic gel that governs vascular permeability and facilitates the mechanical transduction of shear stress (184, 185). This glycocalyx is an integral part of the glomerular filtration barrier, and MRAs lower matrix metalloproteinase (MMP) activity, curtailing GEnGlx damage. Consequently, this reduces glomerular permeability and proteinuria in individuals with diabetes (183).

Since 2013, clinical trials involving finerenone, such as The Mineralocorticoid Receptor Antagonist Tolerability Study (ARTS), have unveiled promising clinical outcomes (**Supplementary Table 1**). These findings have heralded a new era in DKD treatment. Notably, the recently published FIDELIO-DKD and FIGARO-DKD studies serve as complementary trials that assess the renal and cardiac benefits of finerenone in DKD patients. Not only does finerenone significantly attenuate the decline in eGFR and improve albuminuria, but it also substantially reduces the risk of DKD progression. Furthermore, the compound significantly diminishes the incidence of new-onset heart failure and decreases the risk of cardiovascular death and non-fatal myocardial infarction (186, 187). The approval of finerenone marks the advent of the “triple therapy” era in DKD treatment.

### 4.4 Potential therapeutic targets for the treatment of DKD

High blood glucose levels promote release of HMGB1, an endogenous ligand of TLRs and the receptor for RAGE, from tubular epithelial cells and glomerular podocytes. HMGB1 consists of an A box and a B box, with the B box binding to TLR2, TLR4, and RAGE, activating the NF-κB signaling pathway and inducing pro-inflammatory responses. Conversely, the A box can dampen the production of pro-inflammatory cytokines stimulated by HMGB1 (188–193). Presently, therapeutic strategies targeting HMGB1 largely center around soluble receptors for advanced glycation end products (esRAGE) and recombinant HMGB1 A box (190, 194). esRAGE, a splice variant of the RAGE gene, can sequester extracellular RAGE ligands, inhibiting RAGE activation on the cell surface. It hinders the binding of HMGB1 to RAGE, TLR2, and TLR4. A clinical study demonstrated that esRAGE overexpression can reduce proteinuria, glomerular disease, and diabetic kidney injury (195). Moreover, TLR4 has close associations with tubulointerstitial inflammation in DKD. Enhanced TLR4 activity triggers the NF-κB signaling pathway and elicits inflammatory responses. TLR4 is implicated in mediating tubulointerstitial inflammation in DKD. Consequently, inhibition of the TLR4/NF-κB pathway may offer a novel therapeutic avenue for DKD (46). TLR2 and TLR4 are highly expressed in renal tubules and their levels have been linked to interstitial macrophage infiltration, HbA1c levels, and eGFR at the time of renal biopsy (48). Thus, targeting HMGB1 through recombinant A box or TLR inhibition/blockade could potentially mitigate proteinuria, glomerular damage, interstitial fibrosis, and renal inflammation in DKD, offering a prospective therapeutic approach (196, 197).

Klotho protein, a single-pass transmembrane protein primarily found in renal tubular epithelial cells, confers kidney protection by exerting anti-aging, anti-inflammatory, anti-oxidative stress, and anti-fibrotic effects (4, 105). Clinical evidence from the PREDIAN trial highlights that the combination of spironolactone with RAS blockers prevents the downregulation of Klotho protein. Additionally, this combination treatment suppresses the expression of TNF-α and TNF-like weak inducer of apoptosis (TWEAK) in renal tubular epithelial cells as adjunctive therapy (141, 198–200). Notably, serum levels of soluble Klotho (sKlotho) and NGAL exhibit a significant negative correlation in DKD patients with varying degrees of albuminuria. These markers could potentially serve as predictive biomarkers for DKD progression in the future (4, 105).

Phosphorylation of OPN triggers the infiltration of macrophages into the renal interstitium Phosphorylation of OPN triggers the infiltration of macrophages into the renal interstitium (38). OPN is prominently expressed in various renal tubular segments, including proximal tubules, distal tubules, medullary thick ascending limbs, and some collecting duct epithelial cells. As a functional non-collagenous protein, OPN serves roles in cell adhesion and recruitment. It induces macrophage chemotaxis, modulates the NO signaling pathway, and mediates the release of inflammatory and fibrotic factors (such as Ang II, TGF-β, endothelin-1) implicated in the pathogenesis of DKD, contributing to tubulointerstitial damage (201, 202). Compared to MCP-1, OPN exhibits a closer pathological relationship with tubular lesions, emerging as a significant therapeutic target for addressing tubulointerstitial inflammation in DKD.

The NF-κB signaling pathway holds key importance in the development of tubular inflammation. Research emphasis lies in inhibiting NF-κB activity and regulating downstream pro-inflammatory cytokines. Related drugs include BAY 11-7082, NF-κB inhibitor PDTC, Rho kinase inhibitors, and Triptolide (138, 203–207). Inhibiting downstream pro-inflammatory factors of NF-κB reduces ROS generation, macrophage infiltration, and related inflammatory factor expression in kidneys, subsequently mitigating renal interstitial damage (208–211). For example, TNF-related apoptosis-inducing ligand (TRAIL) causes apoptosis of renal tubular epithelial cells under high glucose levels and pro-inflammatory cytokine influence, which correlates with the extent of tubular atrophy, interstitial fibrosis, and inflammation (212–214). CCL5 markedly increases in renal tubular epithelial cells and directly correlates with proteinuria and interstitial cell infiltration (93, 215, 216). CXCL16, a pro-inflammatory cytokine, is elevated in renal tubular epithelial and interstitial cells in DKD. By activating the TLR4/NF-κB pathway, CXCL16 exacerbates DKD, inducing inflammatory cell infiltration and heightened cell apoptosis (217–219). Recent research has spotlighted the role of IL-17A in propelling renal inflammation and DKD progression. Interleukin-17A (IL-17A), an immune-related cytokine, spurs inflammatory and immune responses (220). Binding to its receptor IL-17RA, IL-17A triggers tubulointerstitial cell activation, prompts inflammatory cell infiltration, and elicits tubular injury (221). Therefore, inhibiting the CXCL16 and IL-17A/IL-17RA pathways potentially unveils a new therapeutic strategy for DKD.

TGF-α1, β2, and β3 are the most potent promoters of ECM accumulation, with the Smad pathway being pivotal in TGF-β signaling within DKD. This pathway plays a critical role in the accumulation of renal intrinsic cells and ECM molecules (222, 223). Suppression of TGF-β-mediated effects and Smad2 phosphorylation can alleviate renal interstitial fibrosis, tubular atrophy, and improve inflammatory cell infiltration and fibroblast proliferation in mice (224–227). BMP-7, a natural TGF-β antagonist, exhibits robust renal protective functions, effectively reversing renal fibrosis across various animal models (76, 228). However, clinical trials assessing BMP-7 for kidney diseases remain unreported to date.

In addition, the realm of traditional Chinese medicine (TCM) and herbal medicine has uncovered anti-inflammatory effects. Paeoniflorin, for instance, demonstrates the ability to reduce macrophage infiltration and expression of inflammatory cytokines, enhancing kidney function and ameliorating histological damage in diabetic mice kidneys (229). Tripterygium glycosides exhibit anti-inflammatory properties and effectively prevent oxidative-induced glomerular membrane rupture, consequently reducing proteinuria in glomerulonephritis and impeding DKD progression (230–232). While low-quality evidence suggests that Tripterygium could be a potential complementary therapy for DKD, most of these studies remain confined to the cellular, molecular, and animal levels (**Supplementary Table 2**), with a lack of large-scale prospective studies specifically targeting tubular lesions and high-quality pathological evidence substantiating their efficacy.

These potential therapeutic targets and signaling pathways provide novel insights and avenues for the treatment of DKD. Future research should delve deeper into the therapeutic effects and safety of these targets and pathways.

## 5 Conclusion remarks

In conclusion, the investigation of tubulointerstitial inflammation in DKD has garnered significant attention and importance. Understanding the underlying mechanisms and identifying potential therapeutic targets are crucial for the development of effective treatment strategies. The recognition of potential targets, including TLR4, HMGB1, MCP-1, OPN, BMP-7, and Klotho protein, along with the pivotal roles played by the NF-κB and TGF-β/Smad signaling pathways, presents promising avenues for targeted interventions. Nevertheless, further in-depth research is imperative to explore tubulointerstitial inflammatory lesions in DKD comprehensively and gather robust evidence supporting these targets and pathways. By prioritizing the management of tubulointerstitial inflammation, we can advance our understanding of DKD, devise innovative strategies for prevention and treatment, and ultimately enhance outcomes and the overall quality of life for individuals impacted by this disease.

## Author contributions

HJ and XT conceived the manuscript and provided supervision. CX drafted the initial manuscript. HJ and XT reviewed and edited the manuscript. XH and SY contributed to manuscript preparation. All authors have read and approved the final version of the manuscript for publication.
