# Supplementary material for: Advances in understanding and treating diabetic kidney disease: focus on tubulointerstitial inflammation mechanisms
Source: Front Endocrinol (Lausanne). 2023 Oct 4;14:1232790. doi: 10.3389/fendo.2023.1232790 (PMC10583558; doi:10.3389/fendo.2023.1232790)
Supplement: Supplementary file 1 [file Table_1.pdf]

Supplementary Material

Advances in Understanding and Treating Diabetic Kidney Disease: Focus on Tubulointerstitial Inflammation Mechanisms

Chengren Xu*，Xiaowen Ha，Shufen Yang

*** Correspondence:** Xuefei Tian：xuefei.tian@yale.edu

Hong Jiang：jangh-yt@163.com

1 Supplementary Figures and Tables

1.1 Supplementary Tables

Supplementary table 1. Commonly used drug research

Name of drug	Group	Target	Mechanism	Results	Author and publication date
RASi					
captopril	Self-control before and after treatment	Inhibit ACE	Reduces glomerular perfusion and pressure and improves Hemodynamics	lower blood pressure and protect kidney function in patients with diabetic nephropathy.	S Björck et al,1986[137]
Losartan	Losartan vs. placebo		Disruption of Ras inhibits NF-KB activity, which in turn inhibits MCP-1 gene	significant renal benefits and overall tolerability was good	B M Brenner et al.2001[139]

Irbesartan	Irbesartan vs.<br><br>amlodipine vs. placebo	Inhibit Ang II	expression and macrophage infiltration	Effective prevention of renal disease progression	E J Lewis et al. 2001[140]
**SGLT-2i**					
dapagliflozin	Glucose+mannitol vs<br><br>Glucose+dapagliflozin	Inhibit SGLT 2	(1) High glucose-induced autophagic flux reduction, via increased AMPK activity and mTOR suppression;(2) inflammatory alterations due to<br>• NF-κB pathway suppression.	Improve inflammation	Xu, J.et al.2021[146]
Dapagliflozin	normal vehicle group vs diabetic group vs diabetic+ DAPA (0.75 /1.5/3 mg/kg).		decreased SGLT2 expression	TNF-α, PEDF, PTX-3, BCL-2 and Bax were improved	Elkazzaz, S. K.et al. 2021[148]
Empagliflozin	glucose vs glucose+ Empagliflozin		decreased SGLT2 expression, the expression of TGF-beta1, accumulation of extracellular matrix proteins (Fibronectin, Collagen IV), as well as (phosphorylated-smad3) P-smad3 were decreased	Protect proximal renal tubular epithelial cells injury of high glucose-induced	Ndibalema, A. R. et al.2020[151]

empagliflozin	glucose vs glucose+ Empagliflozin	Inhibit SGLT 2	High glucose-induced superoxide and hydrogen peroxide generation, oxidative stress-dependent TRAF3IP2 upregulation, NF-κB and p38 MAPK activation, inflammatory cytokine expression (IL-1beta, IL-6, TNF-alpha, and MCP-1), miR-21 induction, MMP2 activation, and RECK suppression. These were inhabited by EMPA	have the potential to treat kidney inflammatory response/fibrosis and nephropathy under chronic hyperglycemic conditions, such as DKD.	Das, N. A. et al.2020[154]
Empagliflozin	Empagliflozin vs. placebo		Inhibition of SGLT2 activity in proximal convoluted tubules, reduction of sodium and glucose reabsorption, improvement of glomerular hyperperfusion, high pressure, and high filtration, increase of expression of hif-1α, improvement of tubulointerstitial injury and fibrosis; The production of hs-CRP, TNF-α, IL-6 and IFN-γ was decreased by targeting the down-regulation of IL-1ß, TLR4 and active NF-κB, and TGFB/Smad3 signaling pathway was blocked to exert anti-	had lower rates of major cardiovascular events and death compared with the placebo.	Bernard Zinman, M.D.et al.2015[160]
Dapagliflozin	Dapagliflozin vs. placebo			the risk of a composite of a sustained decline in the eGFR of at least 50%, end-stage kidney disease, or death from renal or cardiovascular causes was significantly lower than with the placebo.	Heerspink,H.J.L.et al. 2020[155]
Canagliflozin	Canagliflozin vs. placebo			Double the serum creatinine level or reduce the relative risk of death	Bruce Neal, M.B.et al.2019[156]

			inflammatory and anti-fibrotic effects		
Empagliflozin	Empagliflozin vs. placebo	2022 Ongoing			
GLP-1 Agonists					
liraglutide	Self-control before and after treatment	Activate Glp-1	inhibit PKA and NAD(P)H oxidase	increased the expression of NAD(P)H oxidase components, TGF-beta, fibronectin in renal tissues, and urinary albumin excretion	Hendarto, H. et al.2012[162]
exendin-4	Self-control before and after treatment		acted on the GLP-1 receptor, and attenuated the release of pro-inflammatory cytokines from macrophages and ICAM-1 production on glomerular endothelial cells.	ameliorate albuminuria	Kodera, R. et al 2011[161]
exenatide	exenatide vs glimepiride			reduces urinary TGF-beta(1) and type IV collagen excretion and microalbuminuria.	Zhang, H. et al 2012[165]
Dulaglutide	Dulaglutide vs. placebo			the risk of cardiovascular events was lower than placebo	Hertzel C Gerstein et al.2019[158]
Efpeglenatide	Efpeglenatide vs. placebo				Hertzel C Gerstein et al.2021[159]
**MRA**					

Spironolactone	Different dose (1/30 mg/kg/day) of spironolactone vs placebo	Inhibit MR	Antagonism improves proximal tubule integrity by targeting mTOR/S6K1 signaling and redox status independent of changes in blood pressure.	The structure and function of the proximal tubule were improved	Adam T. Whaley-Connell A et al，2012[178]
Finerenone	Finerenone vs placebo vs Spironolactone		Finerenone can increase the bioavailability of NO and decrease the level of superoxide anions by up-regulating SOD activity	At least as effective as spironolactone in decreasing biomarkers for hemodynamic stress, less hyperkalemia, and a decrease in renal function	Bertram Pitt et al.2013[172]
Finerenone	Self-control before and after treatment			Improve endothelial dysfunction, reduce proteinuria	Raquel González-Blázquez et al，2018[180]
Finerenone	Finerenone vs placebo		Blocking, through both genomic and nongenomic actions, improves proximal tubule structure and function, an improvement that would be associated with a reduction in S6K1 phosphorylation activation and oxidative stress	There was a significant reduction in kidney failure, mortality and risk of cardiovascular events	George L. Bakris et al，2020[186]
Finerenone	Finerenone vs placebo			Kidney failure and EGFR decreased continuously by at least 40% from baseline	B. Pitt, G. Filippatos et al，2021[187]

Supplementary table 2. Potential target therapy for DKD

Name of drug	Experimental model	Target	Mechanism	Results	Author and publication date
BAY 11-7082	SD rats with STZ-induced DN	Inhibit the degradation of I KB	By limiting the activation of NF-κb, the levels of inflammatory cytokines such as TNF-α, il-1β, IL-6 and nuclear transcription factor subunit NF-KB P65 were decreased	Reduced macrophage recruitment and production of inflammatory cytokines	Sambasiva Rao Kolati et al，2015[203]
Y-27632	LPS-induced mice	Inhibit Rho-kinase	Rho kinase inhibition reduced NF-kB P65 phosphorylation and nuclear translocation, reduced the expression of chemokines CCL5 and CCL2, and reduced monocyte/macrophage infiltration.	Reduced inflammation and endotoxic kidney damage	Catherine Meyer-Schwesinger et al.2009[204]
PD98059	HK-2	Inhibit MEK	Down-regulation of the P42/p44 MAPK signaling pathway induced by BK and HG decreased the levels of CTGF, TGF-β and collagen I induced by BK, and inhibited the production of IL-6, CCL-2, and IL-2 induced by HG and BK.	Reduce inflammation, anti-fibrosis	Sydney C W Tang et al.2010[37]

Recombinant HMGB1 a box	WT BALB/C mice	Inhibit HMGB1	Blocking the binding of TLR2, TLR4, RAGE and HMGB1	Proteinuria was significantly reduced and glomerular hypertrophy, glomerular hypercellularity, podocyte protein, podocyte injury, macrophage accumulation, and interstitial fibrosis were relieved	Xiaochen Chen et al.2018[190]
staurosporine	HK-2	Inhibit PKC	The PKC induced by HG was attenuated, the VEGF production induced by HG was blocked, and the production of IL-6, CCL-2, and TGF-β induced by HG was inhibited	Reduce inflammation, anti-fibrosis	Sydney C W Tang et al.2010[37]
Breviscapine	Diabetic rat model	Inhibit MCP-1 receptor	Acts as a receptor antagonist for MCP-1, reducing downstream signaling pathways that induce ROS production and inflammation.	The overexpression of TGF β1 protein observed in the glomeruli and tubulointerstitium of the rats was attenuated	Xing-Xin Xu et al.2013[208]
BMP-7	STZ-induced diabetic mouse model	Inhibit TGF-β receptor	Inhibition of TGF-β receptors significantly reduces renal fibrosis and mRNA levels of key mediators of extracellular matrix deposition in the kidney.	Reduce interstitial fibrosis and inflammatory cell infiltration, reverse renal hypertrophy, improve GFR	Song Wang et al.2003[228]
IN-1130	Rat model of unilateral ureteral obstruction	Inhibit ALK5	The purified kinase domain that inhibits ALK5-mediated Smad3 phosphorylation	Reduced interstitial fibrosis (reduced tubular atrophy, loss,	J-A Moon et al.2006[224]

				and dilation) and inflammatory cell infiltration	
Tripterygium glycosides	STZ-induced DM model	inhibit the HIF-1α and ET-1 expression.	inhibit the HIF-1α and ET-1 expression.	TG can improve kidney damage in diabetic rats and delay the development of diabetic nephropathy	Chen WD et. Al.2015[232]
Paeoniflorin	STZ-induced type 1 diabetic mice model	Inhibit JAK2/STAT3	Inhibition of the JAK2/STAT3 signaling pathway reduced macrophage infiltration and inflammatory factor expression	the functional and histological damage had been attenuated significantly.	Li, X. et al.2018[229]

WT BALB/c: An experimental mouse characterized by albinism and immunodeficiency; STZ :streptozotocin ; LPS: Lipopolysaccharide；SD: Sprague-Dawley; HK-2：human proximal tubular cells
